# Automated Quantitative Assessment of Proteins' Biological Function in Protein Knowledge Bases

**DOI:** 10.1155/2008/897019

**Published:** 2008-06-30

**Authors:** Gabriele Mayr, Günter Lepperdinger, Peter Lackner

**Affiliations:** ^1^Department of Molecular Biology, University Salzburg, Hellbrunner Strasse 34, 5020 Salzburg, Austria; ^2^Institute for Biomedical Aging Research, Austrian Academy of Sciences, Rennweg 10, 6020 Innsbruck, Austria

## Abstract

Primary protein sequence data are archived in databases together with information regarding corresponding biological functions. In this respect, UniProt/Swiss-Prot is currently the most comprehensive collection and it is routinely cross-examined when trying to unravel the biological role of hypothetical proteins. Bioscientists frequently extract single entries and further evaluate those on a subjective basis. In lieu of a standardized procedure for scoring the existing knowledge regarding individual proteins, we here report about a computer-assisted method, which we applied to score the present knowledge about any given Swiss-Prot entry. Applying this quantitative score allows the comparison of proteins with respect to their sequence yet highlights the comprehension of functional data. *pfs* analysis may be also applied for quality control of individual entries or for database management in order to rank entry listings.

## 1. Introduction

Life scientists seek to accumulate knowledge about the distinct functions of biomolecules. Currently, approximately 20000 gene loci with a total of up to 35000 transcripts have been reported for the human genome and transcriptome, respectively [1, 2]. Genomics and proteomics also greatly support efforts of systems biology, which may certainly provide a better understanding of complex biological processes at the organismic level. The prerequisite for this endeavour is to gain as much knowledge as possible about any functional feature in respect to any particular gene product. Therefore, many disciplines in life sciences use protein sequence data for comparative studies in order to assess distinct functional properties in the context of particular biological situations. Beside printed publications, most data acquired have been collected and assembled into a variety of publicly available databases [3–5]. These are generally accepted to represent a common source of knowledge for research related to biochemistry, molecular biology, biomedicine, or systems biology. In addition, to simply archiving data, interdisciplinary efforts are currently being undertaken to fully annotate genomic and proteomic sequence data sets [6]. The overall goal in this context is to increase the general and detailed understanding about genomes and proteomes.

UniProtKB/Swiss-Prot and UniProtKB/TrEMBL store protein primary data and associated biological and biochemical information. These two are often used as the prime source for data mining. In this study, we focused on the knowledge regarding functional aspects of proteins archived in Swiss-Prot because this repository is generally believed to be a high-quality, manually curated protein knowledge base [7]. Moreover, relevant information is primarily extracted from original publications and review articles, and qualified feedback from external experts is also taken into consideration by the curators of Swiss-Prot. By implementing standardized operational procedures, they build up and edit individual entries [8]. Briefly, Swiss-Prot entries contain information concerning literature references, functional descriptions, domain structure, isoforms, and many more selected information regarding the respective protein [3]. Most entries provide a summary of functional aspects as well as other details, such as posttranslational modifications within their *COMMENTS* field. Firm data such as those derived from biochemical analyses are assigned clear-cut attributes. For instance, the various properties of an enzyme are recorded by a *CATALYTIC ACTIVITY* as well as by a *FUNCTION* attribute. Yet, in cases other than enzymes, any other biological function can also be found within the *FUNCTION* attribute. Biologically, relevant information is also reflected in the protein name (*DESCRIPTION* line). Other categories such as *ENZYME REGULATION, PATHWAY, and TISSUE* provide only little further information concerning the protein functional properties than already defined by *FUNCTION OR CATALYTIC ACTIVITY.*

When using computer-assisted methods, the primary structure of proteins can be easily searched for likely regions of resemblance. Annotated knowledge can be adopted by applying automated bioinformatic routines for those sequences, which are not explicitly listed in Swiss-Prot, but however, share remarkable similarity with experimentally well-studied proteins or protein sequence motifs that have previously been characterized in detail [7, 9, 10]. It has now become generally accepted that information available for proteins from closely-related species can be linked to the human proteome. However, complex information regarding experimental data still has to be manually extracted from published literature or databases. In order to enhance this tedious procedure, we here report a novel method, which weighs the quality of an entry with respect to proposed functional properties of proteins. In order to accomplish that, all textual descriptions within Swiss-Prot were scanned for expressions that specify whether experimental analysis regarding the proteins' biological function is yet in progress or whether published results are still insufficient in order to accurately deduce the protein's role with respect to any particular biological mechanism, pathway, or process. The output of our computer-assisted examination resulted in a score for every database entry, which grades the currently existing knowledge and was, therefore, termed the “protein's function score” (*pfs*).

In parallel to the development of the *pfs* procedure, we performed a peer survey. A representative group of trained biologists was asked to manually assess the functional knowledge for 30 randomly selected Swiss-Prot entries. The outcome and feedback guided us when specifically moulding the computer-assisted procedure for the evaluation of currently available information concerning the individual functional properties of proteins. The resulting computational measure was subsequently used for benchmarking of the current knowledge regarding entries in data repositories and libraries. This relation allowed us to incorporate *pfs* into the result list of protein-protein sequence comparisons. This particular feature provides a reliable indication of how well a particular query hit has been previously annotated regarding functional assignments. Precomputed protein's function scores (*pfs*) and online BLASTP searches against *pfs*-annotated Swiss-Prot database are accessible at http://biwww.che.sbg.ac.at/PFS/.

## 2. Methods

### 2.1. Peer Survey

Database entries were randomly selected. 13 peers comprising of one undergraduate student who has been trained in molecular biology, five PhD students, five post doctoral fellows, and three principal investigators working as scientists in the field were asked to validate the information provided with these particular entries and to regard the displayed existing knowledge about the protein as high, medium, or low.

### 2.2. Computing Knowledge Factor-quantitative Evaluation of Database Entries

**Figure 1:**
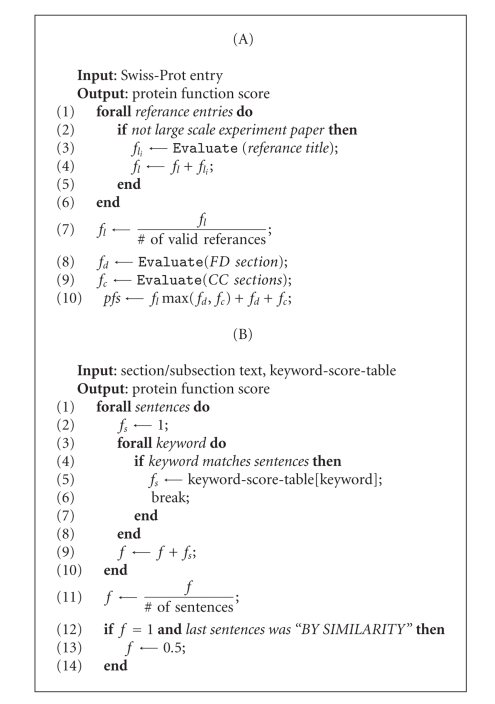
*Computation of *pfs.* (A)* *pfs* was calculated with regard to the validity of bibliographic citations as well as a description and comments section. In line 9, the FUNCTION and CATALYTIC ACTIVITY records were evaluated. In step 2, automated entries resulting from large-scale experimental approaches were detached from further analysis. (B) Section evaluation. In every section, every single sentence was evaluated independently. Lines 12–14 are required in case literature citations contain down-weighting phrases, yet conclusions regarding functional properties of a protein have eventually been made by mere resemblance at the primary sequence level (Swiss-Prot term: “BY SIMILARITY”).

A simple rule was generated in order to yield a solid estimate for the information content of Swiss-Prot: for every single entry, a score was compiled named *pfs*, which is the sum of factors computed for the respective sections of a Swiss-Prot entry, where LITERATURE REFERENCES is denoted *f*_1_, DESCRIPTION = *f*_*d*_, FUNCTION, and CATALYTIC ACTIVITY = *f*_*f*_ (Figure 1). At the beginning of the evaluation procedure, value “1” was assigned to *f*_1_, *f*_*d*_, and *f*_*f*_ as long as the corresponding section displayed any content. Before summing up all increments, which would yield highest possible value *of 3* for *pfs*, all fields were searched for words or phrases of devaluating meanings, which in due course result in the decrease of the relevant factors, *f*_1_, *f*_*d*_, and *f*_*f*_. Terms were selected, which reflect the deliberation of authors to express uncertainty (Table 1). Several of the terms with a specified meaning have been defined by Swiss-Prot [11]. For example, the *DESCRIPTION* or *FUNCTION* fields may contain terms which underline the complete or near inability to assign biological function, such as “unknown,” “not known,” “unnamed,” “hypothetical,” and “uncharacterized.” In cases where the *DESCRIPTION* field contains the term “putative,” *f*_*d*_ is decreased to 0.5. If the *DESCRIPTION* includes “unnamed,” this would result in a zero contribution to *pfs*. In addition to this, a factor was assigned less weight when the entry was qualified as to be deposited “by similarity.” During the computational evaluation, each field was split into single sentences and each sentence was searched for devaluation phrases. When analyzing a sentence, the phrase list was searched from top to bottom. The first match determined the reduction of the respective factor *f*. Validation of literature references was processed as follows: firstly, every single literature reference was evaluated with respect to the same criteria as described for *f*_*d*_ and *f*_*f*_, that is, every single literature reference was searched for keywords, and subsequently, the average raw literature factor was calculated. Exceptionally, Swiss-Prot entries may be the result of automated submissions of genome or expressed sequence tags (ESTs) sequencing data. The accompanying publications rarely address functional questions nor do they experimentally verify bioinformatic analyses via firm biochemical investigations prior to submission to Swiss-Prot. Therefore, Swiss-Prot added a special attribute for large scale experiments, such as genome sequencing or functional genomic approaches. These publication formats were rated zero. Apparently, a large number of publications is often a good reflection of accumulating knowledge. However, *f*_1_ (*pfs* increment for literature) should not overwhelm the value of functionally-related terms as defined before. As a matter of fact, entries for proteins with largely unknown molecular function typically have *f*_*d*_ and *f*_*f*_ values that are low or close to zero. In cases of entries that contain numerous publications, the mere addition of an average raw literature factor to the aforementioned low *f*_*d*_ and *f*_*f*_ would yield a deceptively high *pfs* value. Therefore, the average raw literature factor was compared to the assessment calculated from the sections *DESCRIPTION*, *FUNCTION*, and *CATALYTIC ACTIVITY*, respectively. The average literature factor was factorized by multiplication with the maximum of *f*_*d*_ and *f*_*f*_. The proposed scheme has been built and refined iteratively by reading the content of several hundred Swiss-Prot entries and checking the coherence with the calculated *pfs*. The source code for computing pfs for Swiss-Prot is available upon request.

## 3. Results

### 3.1. Evaluation of Database Entries by Peers

Scientists, who routinely use Swiss-Prot or have been trained to extract information about protein from a variety of data repositories, were asked to evaluate 30 randomly picked Swiss-Prot entries. Eventually, the quality of all data as provided within the Swiss-Prot entry for this particular protein should be regarded as being high, medium, or low (Table 2). In cases, where the biological role or the functional property of the respective proteins is properly described by published data, the cumulative knowledge about this protein was considered high. In cases, where there is still experimental data necessary to clarify the biological function of the protein or the data are purely descriptive, the information content presented in such entries was ranked medium or low. No further sources of information than those provided by Swiss-Prot had been allowed for this particular evaluation. In a few cases, there appeared only little concordance between peer evaluation results (see Table 2, column “maj”). As a lowest degree of consistence, 38% was obtained regarding database entries P01827 and Q9N2B6, respectively. Total agreement was achieved again in two cases, Q57910 and P05484. Random scoring would have resulted in 30% of the entries gaining the lowest possible concordance score (39%) and 90% achieving <62% of consensus, which is in strong contrast what was attained by the peer group. The lowest possible as well as the highest concordance score were much lower, 7% and 50% respectively. Apparently, there seems to be a bias due to personal appreciation in respect to knowledge archived in biological databases. For instance, PI3 assigned grade “high” to two thirds of the entries, while Ph2 accounted the same number of entries as containing little knowledge. Additionally, the peers also had to report one field for every particular database entry, which had the highest impact on her/his individual decision. In 35% of the decisions, the COMMENT-FUNCTION line was considered most important, in 16%, it was the protein's name as provided in the DESCRIPTION line, and in 13%, the reference list was most appealing for the evaluating peers. Lines such as COMMENTS, FEATURES, COMMENTS-CATALYTIC ACTIVITY, CROSS REFERENCES, or KEYWORDS were chosen only in below 10% of the cases of the individual decision making processes. Most interesting in this context, peers rarely pointed out that the decision was decisively influenced by literature citations that are embedded in most Swiss-Prot entries. We would furthermore like to note that peers, who applied subjective decision making criteria when validating the currently archived knowledge about a protein's function, did not raise any objections to grade individual entries according to set quality ratings such as low, medium, or high. This strongly suggested to us that any quantitative measure resulting from a standardized procedure, with particular attention paying to biological function information as contained in individual database entries would be also appreciated.

### 3.2. Computational Evaluation of Database Entries

Quality assessment of all Swiss-Prot entries was done by putting weight on to what is currently known about the biological function(s) of an individual protein. The information is abstracted in the respective sections of a Swiss-Prot entry *LITERATURE REFERENCES*, *DESCRIPTION* and *FUNCTION*, and *CATALYTIC ACTIVITY*. These fields were also highly appreciated by the peers for measuring biological function. Therefore, the text included in these sections was carefully evaluated for particular specifications of down-weighing characterizations as shown in Table 1. Next, the above-mentioned database fields of every single Swiss-Prot entry were examined, and the respective information content was weighed in order to recount and score the currently existing knowledge with regard to the protein's functional properties. As described in the methods section, summing the resulting scores that had been calculated from the database lines equals *pfs* with a maximum value of 3 (Figure 1). The evaluation scheme acts on the assumption that any field contains standardized textual information. In order to ensure that, the procedure relies not only on parsing of the quantity of text contained in every field, we determined whether there is any correlation between word counts of the individual fields reference title, COMMENTS or DESCRIPTION, and *pfs*. When plotting log (number of words) against *pfs*, only a weak correlation of *r* = 0.66 after applying Pearson's formula could be observed (when omitting log scaling, correlation decreases to *r* = 0.42). In addition to this, we observed a rather broad distribution with respect to word count, which makes it hard to believe that *pfs* may be deduced by merely counting words. We furthermore analyzed the degree of consent between peers scoring and *pfs*. Taking the entire data set into account, only a week correlation of *r* = 0.58 could be revealed. When selecting only those cases that resulted in a concordance score of >62%, which was half of the entries, a correlation of *r* = 0.78 was obtained.

As a next step, *pfs* was used to classify all Swiss-Prot entries by sorting them into three categories, representing a “low” (0 < 1), “medium” (1-2), and “high” degree of knowledge (>2) presently available with respect to function and/or biological role (Table 3). Low knowledge results from the fact that the number of publications is low and/or the amount of extracted functional information is minute or currently still unavailable. For proteins with moderate knowledge, either many publications or some evidence of a particular biological function is publicly accessible. Solid knowledge with a high *pfs* is only reflected by well-documented, reliable information concerning biological function, in particular, clear-cut statements without speculative statements or negative expressions concerning function. The *pfs* for all Swiss-Prot entries can be obtained online at http://biwww.che.sbg.ac.at/PFS/.

Due to the fact that this novel quality score reflects commonly available knowledge concerning a protein's function, we next appended *pfs* to the headers of sequence data files, which we subsequently used for sequence comparisons. When performing protein-protein sequence comparisons with the aid of BLASTP as a search algorithm and Swiss-Prot as a data source, now, the result lists displayed not only a generic protein name, but also provided a list with additional notice specifying the functional information content of any particular hit. In this way, the most informative link becomes highlighted, and furthermore, the quality and quantity of functional data, which are available for a set of proteins related to the subject sequence, can be immediately extracted. This service is also freely accessible online at http://biwww.che.sbg.ac.at/PFS/.

## 4. Discussion

Functional annotations of proteins are collected and provided in databases. Although bioinformatic analyses are regarded to provide unprecedented precision and high performance and are thus being employed to specifically define biological mechanisms at the molecular, genomic, and cellular level, most researchers in life science still (like to) read and survey data collections without applying a standardized validation method. Since knowledge regarding the biological function of proteins is not evenly distributed in databases, and standardized bioinformatic procedures, which would allow individual researchers to specifically qualify information provided in knowledge databases, have not been established yet, which allow individual researchers to specifically qualify information provided in knowledge databases, we developed a novel method. Assessment and cross validation of the current knowledge regarding proteins presented in Swiss-Prot by this linguistic analysis yielded results, which can be hardly achieved by any sort of peer evaluation procedure. In line with this, it is also not surprising that only in selected cases a strong correlation between results of the presented peer data and *pfs* characterization became apparent. Moreover, assignment of *pfs* can be easily adjusted to validate other scientific databases, which, for example, accumulate large amounts of textual descriptions or papers. In the context of annotated protein data, *pfs* can now be employed to determine the knowledge status of large sets of proteins or groups of homologous proteins as well as to tentatively assign potential functions to any proteome.

Considering the impact on daily research, we propose that *pfs* is appended to protein identifiers within a sequence comparison result list. In this way, nonexperts in a particular field quickly obtain a ranked listing of knowledge distribution when examining the hit list. In addition, tagging protein identifiers with *pfs* are most helpful for identifying links, which guide one to the most informative database entry. Hence, we regard *pfs* as an important improvement for accelerating data mining strategies and to providing new avenues for evaluating data derived from genomic and proteomic projects. In the near future, this tool may become an invaluable tool for curators of knowledge databases, primarily to earmark entries which are of minor quality, in order to either eliminate the entry or to improve it during a subsequent update.

## Figures and Tables

**Table 1:** *Expressions and corresponding weights used in pfs calculation:* terminology of devaluating meaning frequently used in Swiss-Prot entries, which were applied in (1).

Unknown	0.0
Not known	0.0
Not yet known	0.0
unnamed	0.0
Uncharacterized	0.0
Potential	0.5
Not clear	0.5
Not yet clear	0.5
By similarity	0.5
Putative	0.5
Similar to	0.5
Possible	0.5
Seems to	0.5
Thought to	0.5
Could be	0.5
Uncertain	0.5
Potentially	0.5
Might	0.5
May	0.5
Presumably	0.75
Probably	0.75
Probable	0.75

**Table 2:**
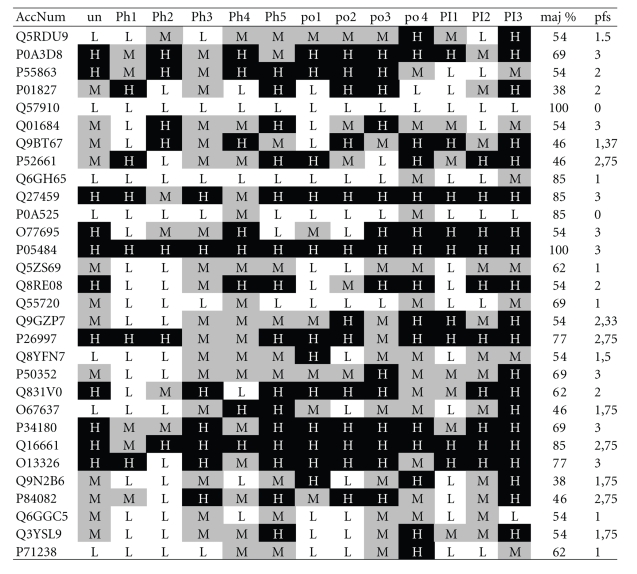
*Peer survey*. 30 randomly picked Swiss-Prot entries (accession number: AccNum) were evaluated by trained biologists (undergraduate: un; PhD student: Ph; postdoctoral fellows: po; principal investigators: PI). Grades given for the provided information, in particular concerning functional properties as exemplified within an entry were “low” (L), “medium” (M) or “high” (H). The concordance of the ranking assigned by the peers is depicted (maj %), pfs: protein function score.

**Table 3:** * pfs analysis of Swiss-Prot entries*. Absolute numbers of individual entries that were grouped according to computed *pfs* value are shown for the most prominent species in Swiss-Prot.

*pfs*						*Species*
*n*	0	<1	1-2	>2	3	
17169	880	956	4973	11240	5238	*Homo sapiens*
13826	441	480	5326	8020	3545	*Mus musculus*
6493	1609	1650	941	3902	2470	*Saccharomyces cerevisiae*
6312	123	138	1710	4464	2278	*Rattus norvegicus*
6065	202	594	2233	3238	1613	*Arabidopsis thaliana*
4402	955	1024	1117	2261	1661	*Eschericia coli*
4272	130	145	2454	1673	995	*Bos taurus*
3072	657	797	1310	965	609	*Caenorhabditis elegans*
2860	753	760	641	1459	940	*Bacillus subtilis*
2612	17	19	580	2013	1031	*Drosophila melanogaster*
2199	49	56	1118	1025	515	*Xenopus laevis*
1935	58	63	1656	216	107	*Pongo pygmaeus*
1837	21	25	597	1215	652	*Gallus gallus*
1774	421	460	1090	224	136	*Haemophilus influenzae*
1652	47	54	900	698	481	*Salmonella typhimurium*
1536	36	43	982	511	211	*Brachydanio rerio*
1420	410	495	770	155	107	*Mycobacterium tuberculosis*
1401	6	20	489	892	412	*Oryza sativa*
1234	1	2	256	976	586	*Sus scrofa*
1226	26	36	744	446	324	*Pseudomonas aeruginosa*
919	3	11	829	79	51	*Yersinia pestis*
**283454**	**14317**	**17142**	**165127**	**101185**	**59443**	**Total**

## Abbreviations

*Pfs*: Protein's function score
BLASTP: Basic local alignment search tool.
